# Effects of acupuncture combined with trunk strengthening training on balance and gait abilities in stroke hemiplegic patients

**DOI:** 10.1097/MD.0000000000037784

**Published:** 2024-07-19

**Authors:** Xiaoping Hong, Shibin Li, Zhuoqin Zhong, Yu Lin, Kunmu Zhang

**Affiliations:** a Department of Rehabilitation, The Second Affiliated Hospital of Fujian University of Traditional Chinese Medicine, Fuzhou, P. R. China; b Department of Rehabilitation, The Affiliated People’s Hospital of Fujian University of Traditional Chinese Medicine, Fuzhou, P. R. China; c Department of Rehabilitation, Fujian Provincial Hospital, Fuzhou, P. R. China.

**Keywords:** acupuncture, balance function, gait ability, stroke, trunk strengthening training

## Abstract

This study aimed to observe the effects of acupuncture combined with trunk strengthening training on balance and gait abilities in stroke hemiplegic patients. Sixty stroke hemiplegic patients were selected and randomly divided into a treatment group and a control group, with 30 patients in each group. The control group received conventional rehabilitation training and trunk strengthening exercises, while the treatment group received acupuncture in addition to the same interventions. Before and after 8 weeks of treatment, patients were assessed using the Holden Functional Ambulation Categories and Berg Balance Scale, and measurements were taken for step length, step width, and gait speed. Prior to treatment, there were no significant differences in Holden scores, Berg scores, step length, step width, or gait speed between the 2 groups (*P* > .05). After 8 weeks of treatment, significant improvements were observed in the aforementioned parameters in both groups (*P* < .05), with the acupuncture group showing significantly greater improvement compared to the control group (*P* < .05). Acupuncture combined with trunk strengthening training can significantly improve balance and gait impairments in stroke hemiplegic patients.

## 1. Introduction

Stroke, also known as apoplexy or cerebrovascular accident, is an acute disorder characterized by cerebral functional impairment due to cerebral vascular disease leading to cerebral blood supply disruption.^[1–3]^ It primarily manifests as unilateral limb paralysis, facial numbness on one side, and paralysis of the upper and lower limbs on the same side. This disease is characterized by a high incidence and disability rate, with approximately 2 million new stroke patients in China each year, among whom 70% to 80% may experience residual limb paralysis.^[4]^ Poststroke symptoms vary depending on the location and extent of brain damage and may encompass impairments in motor, sensory, speech, swallowing, cognitive, and psychological functions.^[5]^ Among these impairments, motor dysfunctions such as balance and gait disturbances are the most common and impactful, potentially resulting in disabilities,^[6]^ and significantly reducing the quality of life for patients.

The mechanism by which stroke patients develop balance or gait problems could be explained that the brain coordinates movement, balance, and gait through a complex interplay of various regions, including the cerebral cortex, cerebellum, basal ganglia, and brainstem. When a stroke occurs, it disrupts the normal functioning of these areas, leading to deficits in motor control and coordination. Balance refers to the ability to automatically adjust the body’s center of gravity to return to a stable position. It plays a crucial role in maintaining daily life and improving walking quality, as it is integral to the completion of various movements in daily activities.^[7]^ In stroke patients, brain damage results in the loss of control from higher neural centers over lower neural centers, leading to abnormal muscle strength and tone, reduced proprioception, impaired muscle coordination, diminished balance, and consequently, abnormal gait patterns and increased risk of falls. These factors collectively diminish the patients’ abilities for daily activities and quality of life.^[8,9]^

The core muscle group, being central to the kinetic chain, significantly influences the restoration of balance function. Research has shown that hemiparetic stroke patients experience reduced core muscle strength, making it challenging to maintain spinal and pelvic stability and resist gravity during movement, ultimately leading to incomplete muscle activation.^[10–12]^ Consequently, it becomes difficult to maintain bodily stability throughout the gait cycle, affecting posture control and weight shifting. Core stability forms the foundation for lower limb movement, thus disruptions in it can lead to balance and gait dysfunctions.^[10]^ Among poststroke impairments, balance and gait dysfunctions have the most adverse impact on patients’ lives. To enhance overall recovery outcomes, particularly in balance and gait, efforts must be directed towards achieving a higher functional level. This not only shortens the treatment period but also mitigates the impact of functional impairments on patients’ lives.^[13]^

Neurological function rehabilitation is currently a focal point in stroke recovery treatment, playing a significant role in improving poststroke functional impairments and enhancing the quality of life.^[14]^ The physiological basis of stroke rehabilitation lies in the plasticity of the nervous system. Repeated training of the paralyzed limbs promotes the transmission of information impulses to the neural centers, stimulates the motor cortex of the brain, and expands its governing areas. This approach is beneficial for rebuilding the function of relatively intact residual neurons and reestablishing normal movement patterns, thereby facilitating the restoration of patients’ motor abilities.^[15]^

Research has demonstrated that rehabilitation therapy during the stroke recovery period has a favorable effect on cortical functional reorganization. After rehabilitation therapy, there is a noticeable increase in cerebral arterial blood flow velocity, yielding more pronounced outcomes compared to drug therapy.^[16]^

Acupuncture, as a traditional treatment method in Chinese medicine, plays a significant role in neurological rehabilitation therapy. It serves not only as a complementary and alternative therapy for stroke rehabilitation but also as a promising preventive strategy in stroke, inducing cerebral ischemic tolerance. As a result, it has been gaining increasing attention in stroke recovery.^[17–19]^ In this study, by combining acupuncture and trunk strengthening training with conventional rehabilitation exercises, we aimed to address functional impairments in poststroke hemiparetic patients. This approach aimed to enhance trunk balance control and consequently improve walking and balance functions, resulting in satisfactory therapeutic outcomes.

## 2. Material and methods

### 2.1. Study subjects

A total of 60 stroke hemiplegic patients admitted to our department were selected as study subjects. The inclusion criteria were as follows: all patients had experienced a first-time stroke and met the diagnostic criteria for stroke established by the 4th National Cerebrovascular Disease Conference,^[20]^ confirmed by head CT or MRI scans; onset of stroke occurred within <2 months, with stable vital signs and clear consciousness; presence of limb functional impairments, and willingness to actively cooperate during treatment; informed consent for participation in this study. Exclusion criteria were: disease duration exceeding 2 months; presence of bone, joint, or muscle disorders; disease deterioration with new cerebral infarctions or hemorrhagic lesions; history of myocardial infarction within the past month, significant impairment or failure of important organs such as heart, liver, or kidneys; severe cognitive and communication impairments, among others. Using a random number table, the selected patients were divided into a treatment group and a control group. General characteristics and disease conditions of the 2 groups are detailed in Table 1. Statistical analysis of the data in the table revealed no significant differences between the groups (*P* > .05), ensuring comparability.

**Table 1:** Comparison of general characteristics between 2 group.

Group	N	Gender (n)		Age	Disease duration	Nature of lesion (n)		Hemiplegic side (n)	
		Male	Female	($\bar{X}\pm S$, y)	($\bar{X}\pm S$, d)	ICH	CI	Left	Right
Treatment group	30	22	8	54.63 ± 4.24	28.50 ± 3.17	12	18	17	13
Control group	30	23	7	55.00 ± 3.79	28.77 ± 3.98	11	19	19	11

**Table 2:** The detailed methods of trunk strengthening training.

Supine position	Sitting position	Transition from sitting to standing position
Passive chest movement training	Sitting pelvic separation movement training	Forward body inclination training
Abdominal muscle control training	Sitting weight shift training	Upright trunk movement training
Bridging exercise bridging exercise	Trunk spasm inhibition training	Upright pelvic anterior and posterior tilt training
Lower trunk flexion and rotation training	Sitting forward and backward movement training	Selective trunk and hip joint movement training
Trunk rotation control training	Selective lower trunk flexion training	Sitting and standing balance response training
Rolling over training	Active trunk gravity-resisted side bending training	
Relieve trunk spasms, enhance stability training		

### 2.2. Treatment methods

All patients were required to take medications prescribed for the underlying condition.

#### 2.2.1. Control group

Rehabilitation training and trunk strengthening exercises were employed in the control group. The following treatments were administered for 45 minutes each session, once daily, 6 days a week, for a total of 8 weeks.

(1) *Rehabilitation training*: Techniques such as the Bobath method, Rood approach, proprioceptive neuromuscular facilitation (PNF), and motor relearning were utilized. This included range of motion training, bed turning and sitting training, standing and walking training, upper limb joint control training, and activities of daily living training (e.g., dressing, toileting, brushing teeth, eating).^[21]^ The detailed training methods were as follows:
  *Bobath method*: Techniques used in Bobath method include the weight shifting, facilitation of postural control, inhibition of abnormal reflexes, and guided movement sequences.
  *Rood method*: Techniques used in Rood approach include the light touch, icing, brushing, tapping, and joint compression to modulate muscle tone and promote desired motor responses.
  *PNF*: Techniques used in PNF include D1 flexion and extension patterns, D2 flexion and extension patterns, rhythmic initiation, rhythmic stabilization, and contract-relax stretching.
(2) *Trunk strengthening training*: The detailed training methods were shown in Table 2.

#### 2.2.2. Treatment group

The treatment group received acupuncture, rehabilitation training, and trunk strengthening exercises.

1. *Acupuncture*: Referring to “Acupuncture Treatment Principles”^[22]^ and clinical experience, acupuncture was applied primarily to the healthy side’s motor area, combined with points related to foot sensation and the 4 Seas. For the lower limbs, acupuncture mainly targeted points along the Gallbladder Meridian, such as GB30 (Huantiao), GB31 (Fengshi), GB37 (Guangming), GB39 (Xuanzhong), GB40 (Qixu), ST44 (Neiting), ST36 (Zusanli), and SP6 (Sanyinjiao). Mild reinforcing and reducing needling techniques were employed, once a day, 30 minutes per session, 6 sessions per week, for 8 weeks.
2. *Rehabilitation training*: Same as the control group.
3. *Trunk strengthening training*: Same as the control group.

### 2.3. Observation indices and methods

#### 2.3.1. Observation indices

1. *Walking ability assessment*: The Holden Functional Ambulation Classification scale was used for assessment.^[23]^
2. *Balance function assessment*: The Berg Balance Scale was utilized for assessment.^[24]^ This scale comprises 14 scoring items, including standing up, sitting down, and independent standing. Each item is scored from 0 to 4 points, with a total possible score of 56. Higher scores indicate better balance function, while scores below 40 suggest a risk of falling.
3. *Gait analysis*: The commonly used footprint analysis method was employed.^[25]^ Time-distance parameters during walking were measured and recorded. Patients were required to walk independently or with the assistance of a cane along a 10-meter pathway. Average step length, step width, and gait speed were measured and recorded 3 times, with the average value taken.

### 2.4. Statistical analysis

Statistical software SPSS 17.0 was used for data analysis. Descriptive statistics, represented as means and standard deviations, were used for continuous data. Analysis of variance was used for comparison of means among multiple groups, and *t*-tests were used for inter-group comparisons.

## 3. Results

### 3.1. Holden and Berg scores

Comparing the Holden and Berg scores of the treatment and control groups before treatment, no statistically significant differences were observed (*P* > .05), indicating comparability between the 2 groups. After treatment, the differences in Holden and Berg scores between the 2 groups were statistically significant (*P* < .05), indicating that the treatment group outperformed the control group in improving walking ability and balance function. Refer to Table 3.

**Table 3:** Comparison of Holden and Berg scores before and after treatment in 2 groups.

Group	Cases	Holden score		Berg score	
		Before treatment	After treatment	Before treatment	After treatment
Control group	30	0.84 ± 0.07	2.00 ± 0.10	1.66 ± 0.11	3.03 ± 0.07
Treatment group	30	0.85 ± 0.06	2.66 ± 0.16	1.68 ± 0.10	3.66 ± 0.10*^,^†

* *P* < .05, compared to before treatment.

† *P* < .05, compared to the control group.

### 3.2. Gait analysis

Before treatment, there were no statistically significant differences in gait parameters (average step length, step width, gait speed) between the 2 groups (*P* > .05), indicating comparability between the groups. After treatment, both groups showed improved gait parameter scores compared to their respective pretreatment scores, and the differences within each group before and after treatment were statistically significant (*P* < .05). Furthermore, the treatment group demonstrated greater improvement than the control group (*P* < .05) as shown in Table 4.

**Table 4:** Comparison of gait parameters before and after treatment in 2 groups.

Item	Control group		Treatment group	
	Pretreatment	Posttreatment	Pretreatment	Posttreatment
Step length (cm)	31.06 ± 0.73	36.18 ± 0.53	31.52 ± 0.92	38.39 ± 0.50*^,^†
Step width (cm)	7.47 ± 0.42	9.40 ± 0.37	7.44 ± 0.42	8.44 ± 0.40*^,^†
Gait speed (cm/s)	30.64 ± 0.43	36.44 ± 0.42	30.57 ± 0.34	38.32 ± 0.31*^,^†

* *P* < .05, compared to before treatment.

† *P* < .05, compared to the control group.

## 4. Discussion

The trunk is capable of flexion, extension, lateral bending, and rotation movements. The main muscles responsible for trunk movement or control are the extensor muscles of the back and the abdominal muscles. Developmental sequence in humans suggests that extension control of the trunk precedes flexion control. For instance, in infants, the extensor muscles develop earlier and are stronger than the abdominal muscles, which delays the ability of infants to sit up directly from a supine position. They tend to roll over first, then sit up from a prone position, often assuming a position of back extension while sitting, and displaying lumbar lordosis and knee hyperextension while standing, along with widened steps and abducted shoulders during walking. The weakened trunk control ability in stroke hemiplegic patients resembles the conditions observed in the motor development of normal infants and toddlers.

In previous rehabilitation concepts, it was common to initiate proximal-to-distal limb function training only after achieving a certain level of core motor function. Often, methods like the Bobath ball and one-on-one therapist-patient training were used, but these approaches lacked objective indicators and specific targeting in rehabilitation. The synchronous functional training of the early trunk was frequently neglected. In clinical practice, it was noted that when lower limb muscle strength of patients reached a certain level of recovery, many patients’ family members would assist them in walking practice. However, this often led to the development of a typical hemiparetic gait, resulting in abnormal trunk postures and negatively impacting limb and balance function rehabilitation.

In this study, trunk strengthening training was added on top of basic rehabilitation treatment. This enabled patients to engage in spine and hip rotational training while maintaining trunk stability. This training included elongating the spine, forward and backward bending, left and right lateral bending, and multidirectional pattern training. These exercises directly targeted training of the muscles of the lumbar back and the lumbar-sacral region, leading to improved trunk stability and control ability. Additionally, this approach accelerated hip, knee, and ankle joint separation movements, disrupted abnormal postures and movement patterns, normalized muscle strength and tone, and promoted the appearance of normal movement patterns.

The results of this study indicate that trunk strengthening combined with basic rehabilitation training can improve the walking and balance functions of stroke hemiplegic patients, with even better effects observed in the acupuncture group. Acupuncture, as a traditional treatment method in traditional Chinese medicine, holds significant value in the recovery of various functions in stroke patients, including motor function of the affected limbs, sensory function, speech function, and activities of daily living.^[26]^ Research has indicated that acupuncture treatment can improve cerebral blood circulation in stroke patients, regulate meridians to promote blood and energy flow, stimulate electrical activity in the brain cortex, enhance peripheral sensory input to brain cells from the affected limbs, and induce or inhibit muscle tone. Different acupuncture techniques can activate motor and sensory functions, facilitating the recovery of function in hemiplegic limbs.^[27]^ Huang et al^[28]^ found that acupuncture can significantly improve static balance in stroke patients with lower Brunnstrom stages, while its effect on balance function is limited in patients with higher Brunnstrom stages. The results of this study show that acupuncture combined with trunk strengthening training can effectively improve balance function in stroke hemiplegic patients, which may be related to the functional reorganization of synapses in different brain regions facilitated by acupuncture, contributing to balance coordination.

Despite the valuable insights gained from this study, several limitations should be acknowledged. Firstly, the sample size in our study was relatively small, which may limit the generalizability of our findings to broader populations. Additionally, this study lacked objective indicators such as blood flow. Future studies employing larger sample sizes and objective indicators are warranted to validate and extend our findings.

## 5. Conclusion

In conclusion, the combination of acupuncture and trunk strengthening training improves the walking and balance functions of stroke hemiplegic patients, yielding significant therapeutic effects and warranting clinical promotion. The optimization of acupuncture and rehabilitation training, timing and selection of different treatment methods, and further enhancement of clinical efficacy represent crucial aspects for future clinical research.

## Acknowledgments

This work was financially supported by the Natural Science Foundation of Fujian Province (2023J01818, 2023J01837), the Middle-aged Backbone Project of the Fujian Provincial Health Commission (2023GGA073), and the National Natural Science Foundation of China (81904316).

## Author contributions

**Data curation:** Zhuoqin Zhong.

**Formal analysis:** Yu Lin.

**Funding acquisition:** Xiaoping Hong, Shibin Li, Kunmu Zhang.

**Investigation:** Zhuoqin Zhong.

**Methodology:** Yu Lin.

**Project administration:** Xiaoping Hong, Shibin Li.

**Supervision:** Kunmu Zhang.

**Writing – original draft:** Xiaoping Hong.

**Writing – review & editing:** Kunmu Zhang.
